# The Effect of Curfew on the General Mental Well-Being of the Population in Saudi Arabia After the COVID-19 Outbreak

**DOI:** 10.7759/cureus.20540

**Published:** 2021-12-20

**Authors:** Abdullah K Alhwimani, Mohamed R Elzahrani, Ahmed S Hilabi, Ghazi A Alghamdi, Yazeed R Elzahrany, Mahmoud H Sami, Mohamed M Ouda

**Affiliations:** 1 College of Medicine, King Saud bin Abdulaziz University for Health Sciences, Riyadh, SAU; 2 Family Medicine, Prince Sultan Military Medical City - Ministry of Defense, Riyadh, SAU; 3 Psychiatry, King Abdullah Specialist Children Hospital - Ministry of National Guard Health Affairs, Riyadh, SAU

**Keywords:** saudi arabia, mental well-being, curfew, world pandemic, covid-19

## Abstract

Introduction

Mental well-being is an essential aspect of general health. Assessing mental well-being is crucial to leading a healthy life. The global population is, presumptively, affected at a ratio of one out of four individuals with a mental or neurological disorder. This further emphasized the importance of the financial, social, and health implications that ensue.

Methods and materials

Data collection was performed using the symptoms checklist-90 (SCL-90) survey between March and April of 2021. The data collected included demographic data as well as nine domains that include some of the most common psychiatric symptoms. The collected questionnaires were analyzed using the Kolmogorov-Smirnov and Shapiro-Wilk tests. Non-parametric tests were utilized, as the SCL-90 dimensions and global index scores follow a non-normal distribution.

Results

The questionnaire yielded 387 responses. Females comprised the majority of the participants. The most prevalent symptom described as extremely common by females was waking up early in the morning. The most commonly described symptom described as not at all by females was hearing words that others do not hear. There was no statistical difference in mental well-being between males and females. Older participants (>40 years old) had better mental well-being in comparison to their younger counterparts.

Conclusion

During the fallout of the coronavirus disease 2019 (COVID-19) pandemic, much attention and resources were allocated toward the physical aspect of the pandemic, yet the psychological implications must not be understated. Multiple variables, such as age, marital status, and unemployment, may impact the mental well-being of the population and must be further assessed.

## Introduction

Mental well-being is an essential factor that should be monitored and appreciated due to its strong effect on many aspects of people’s lives. According to the World Health Organization (WHO), one in four people will be affected by a mental or neurological disorder at some point in their life, which places mental disorders among the leading causes of ill health and disability worldwide [1]. According to the American National Institute of Mental Health (NIMH), the leading cause of disability in the United States is neuropsychiatric disorders [2]. After the coronavirus disease 2019 (COVID-19) outbreak, the world took strict and pivotal actions to decrease harm and save lives from such a pandemic. 

Those actions were detrimental to prevent the spread, but adopting a new lifestyle and following such strict regulations by people worldwide is not easy. The lack of social contact and connectivity caused by the curfew adversely affected the population’s mental wellbeing. For patients with conditions such as post-traumatic stress disorder (PTSD) and anxiety, socializing is beneficial, and the lack of socializing is especially harmful to them. These findings are in line with previous pandemics such as severe acute respiratory syndrome (SARS) and Middle East respiratory syndrome coronavirus (MERS-CoV) [3]. The chances that people will develop disorders such as depression and other mental illnesses due to the new lifestyle they have to adapt to are high and require our attention. For instance, in Saudi Arabia, the local statistics before the COVID-19 pandemic showed that depression is common among 15.3-22% of primary care patients and 17-46% of the Saudi population [4]. These statistics raised a question about the mental well-being of the Saudi population, especially after putting in mind the new factors affecting mental health such as the curfew implemented by the Saudi ministry of health to fight the pandemic.

Moreover, the physical inactivity associated with curfew and social isolation may affect people with specific hypertension, diabetes, and dyslipidemia [5]. According to a study conducted in Saudi Arabia among Saudi female university students, a regimen of improved physical activity for three weeks yielded an improved score in the Insomnia Severity Index, the Beck Depression Inventory, and the Attention Span Test, suggesting that activity can be beneficial in managing insomnia, depression, and attention deficit [6]. Therefore, the opposite effect may be observed due to social isolation, curfew, and less physical activity. 

Also, estimating the percentage of people affected mentally after the lockdown will help primary health care providers, such as Family and Community medicine physicians, to better appreciate the impact of the COVID-19 curfew on their patients’ mental well-being. In other words, a better understanding of the population’s mental status will help the medical field to better adapt to changes that might occur after the COVID-19 outbreak regarding mental illness. No study, to our knowledge, addresses the impact of COVID-19 on the Saudi population’s mental well-being. Therefore, a study addressing this topic is required in Saudi Arabia.

## Materials and methods

Setting and participants

This research used a descriptive cross-sectional study design inclusive of the general Saudi adult population where all the data were collected at one point in time. The study received institutional review board approval on March 6, 2021, at King Abdullah International Medical Research Center, Riyadh, Saudi Arabia (IRBC/0509/21). The study was conducted digitally, targeting Saudi nationals above the age of 18 between the dates of March to April of 2021. The yielded responses were 387 questionnaires.

Questionnaire and data collection

All willing participants above 18 years who spoke either English or Arabic were invited. An informed consent form was filled before accessing the questionnaire. Convenience sampling was applied during data collection. A pre-validated questionnaire was acquired and dissipated. The symptoms checklist 90 (SCL-90)-revised is a self-administered psychometric survey that has been constructed to assess a wide range of psychological symptoms. The questionnaire comprised 90 questions describing symptoms. These symptoms are then grouped into nine main domains that represent major psychiatric conditions including somatization, obsessive-compulsive, interpersonal sensitivity, depression, anxiety, hostility, phobic anxiety, paranoid ideation, and psychoticism. The reliability of the questionnaire was validated and checked to be reliable through multiple studies. The Arabic validated version has been used in this research. Data were collected from March 2021 to April 2021. The first section concerns the demographic data, including gender, age, marital status, employment, educational attainment, and place of residence.

Statistical analysis

Categorical variables were presented using counts and proportions (%) while continuous variables were summarized using mean and standard deviation. SCL-90 dimensions and global index were compared with the socio-demographic characteristics of the participants by using the Mann-Whitney Z-test. P <0.05 was considered statistically significant. Normality, statistical interactions, and collinearity (i.e., variance inflation factor) were also assessed with the Kolmogorov-Smirnov and Shapiro-Wilk tests. SCL-90 dimensions and global index scores follow a non-normal distribution. Thus, non-parametric tests were applied. All data analyses were performed using Statistical Packages for Software Sciences (SPSS) version 26 (IBM Corp., Armonk, NY).

## Results

A total of 387 responses was obtained from our survey. Table 1 describes the socio-demographic characteristics of participants. The most common age category was 18-30 years old (36.7%), with females dominating males (73.1% vs. 26.9%); nearly 60% were married. Most of the respondents were professionals (78.6%), and nearly two-thirds (63.3%) were not working. In addition, 66.7% were living in the Central region. The most prevalent symptom described as extremely common by females was waking up early in the morning (18%) while the most commonly described symptom described as not at all by females was hearing words that others do not hear (85%).

**Table 1:** Socio-demographic characteristics of participants (n=387)

Study variables	N (%)
Age group	
18 – 30 years	142 (36.7%)
31 – 40 years	53 (13.7%)
41 – 50 years	80 (20.7%)
51 – 60 years	85 (22.0%)
>60 years	27 (07.0%)
Gender	
Male	104 (26.9%)
Female	283 (73.1%)
Marital status	
Unmarried	159 (41.1%)
Married	228 (58.9%)
Educational level	
High school or below	83 (21.4%)
Bachelor or higher	304 (78.6%)
Employment status	
Employed	142 (36.7%)
Unemployed	245 (63.3%)
Place of residence	
Central region	258 (66.7%)
Western region	129 (33.3%)

The descriptive statistics of the SCL-90 symptom dimensions and global index were given in Table 2. It was revealed that the highest mean score has been observed in the dimension of obsessive-compulsive (mean score: 1.25) and depression (mean score: 1.25) followed by interpersonal sensitivity (mean score: 1.17) while the least mean score was observed in psychoticism dimension (mean score: 0.82).

**Table 2:** Descriptive statistics of SCL-90 symptom dimensions and global index (n=387) SCL-90: symptoms checklist-90

SCL-90 variables	Mean ± SD
Somatization	1.08 ± 0.71
Obsessive-compulsive	1.25 ± 0.70
Interpersonal sensitivity	1.17 ± 0.79
Depression	1.25 ± 0.82
Anxiety	1.03 ± 0.78
Hostility	0.89 ± 0.72
Phobic anxiety	0.85 ± 0.78
Paranoid ideation	1.04 ± 0.80
Psychoticism	0.82 ± 0.73
Global Severity Index	1.09 ± 0.67

The descriptive statistics of the SCL-90 symptom dimensions and global index are given in Table 3. It was revealed that the highest mean score has been observed in the dimension of obsessive-compulsive (mean score: 1.25) and depression (mean score: 1.25) followed by interpersonal sensitivity (mean score: 1.17) while the least mean score was observed in psychoticism dimension (mean score: 0.82).

**Table 3:** Differences in mental well-being according to age group (n=387) § P-value has been calculated using the Mann-Whitney Z-test. ** Significant at the p<0.05 level

SCL-90 variables	Age ≤40 years Mean ± SD	Age >40 years Mean ± SD	Z-test	P-value ^§^
Somatization	1.11 ± 0.70	1.06 ± 0.73	0.662	0.508
Obsessive-compulsive	1.38 ± 0.74	1.12 ± 0.64	3.321	0.001 **
Interpersonal sensitivity	1.35 ± 0.85	0.99 ± 0.69	4.266	<0.001 **
Depression	1.43 ± 0.85	1.07 ± 0.74	4.276	<0.001 **
Anxiety	1.18 ± 0.83	0.88 ± 0.69	3.567	<0.001 **
Hostility	1.09 ± 0.79	0.68 ± 0.56	5.197	<0.001 **
Phobic anxiety	0.90 ± 0.85	0.79 ± 0.69	0.566	0.571
Paranoid ideation	1.29 ± 0.85	0.79 ± 0.66	6.048	<0.001 **
Psychoticism	0.99 ± 0.78	0.63 ± 0.63	4.563	<0.001 **
Global Severity Index	1.23 ± 0.71	0.96 ± 0.61	3.781	<0.001 **

When measuring the differences in mental well-being based on SCL-90 symptom questionnaires, it was revealed that the mean score of obsessive-compulsive (Z=0.662; p=0.001), interpersonal sensitivity (Z=4.266; p<0.001), depression (Z=4.276; p<0.001), anxiety (Z=3.567; p<0.001), hostility (Z=5.197; p<0.001), paranoid ideation (Z=6.048; p<0.001), psychoticism (Z=4.563; p<0.001), and global severity index (Z=3.781; p<0.0010) were statistically significantly higher in the age group ≤40 years old.

In Table 4, the differences in the mental well-being scores of somatization, obsessive-compulsive, interpersonal sensitivity, depression, anxiety, hostility, phobic anxiety, paranoid ideation, psychoticism, and global severity index did not reach statistical significance (all p>0.05).

**Table 4:** Differences in mental well-being according to gender (n=387) § P-value has been calculated using the Mann-Whitney Z-test.

SCL-90 variables	Male Mean ± SD	Female Mean ± SD	Z-test	P-value ^§^
Somatization	1.01 ± 0.72	1.11 ± 0.71	1.471	0.141
Obsessive-compulsive	1.24 ± 0.72	1.26 ± 0.69	0.174	0.861
Interpersonal sensitivity	1.19 ± 0.78	1.16 ± 0.79	0.490	0.624
Depression	1.26 ± 0.83	1.25 ± 0.82	0.143	0.887
Anxiety	1.08 ± 0.83	1.01 ± 0.76	0.397	0.692
Hostility	1.03 ± 0.81	0.84 ± 0.68	1.844	0.065
Phobic anxiety	0.72 ± 0.68	0.89 ± 0.81	1.770	0.077
Paranoid ideation	1.13 ± 0.82	1.01 ± 0.79	1.359	0.174
Psychoticism	0.92 ± 0.81	0.78 ± 0.70	1.162	0.245
Global Severity Index	1.11 ± 0.69	1.09 ± 0.67	0.194	0.846

In Table 5, unmarried participants had significantly higher mental well-being mean scores in the following dimensions, including obsessive-compulsive (Z=3.093; p=0.002), interpersonal sensitivity (Z=4.238; p<0.001), depression (Z=4.548; p<0.001), anxiety (Z=4.371; p<0.001), hostility (Z=4.844; p<0.001), paranoid ideation (Z=5.517; p<0.001), psychoticism (Z=5.091; p<0.001), and global severity index (Z=4.104; p<0.001).

**Table 5:** Differences in mental well-being according to marital status (n=387) § P-value has been calculated using the Mann-Whitney Z-test. ** Significant at the p<0.05 level

SCL-90 variables	Unmarried Mean ± SD	Married Mean ± SD	Z-test	P-value ^§^
Somatization	1.14 ± 0.70	1.05 ± 0.72	1.368	0.171
Obsessive-compulsive	1.38 ± 0.69	1.16 ± 0.69	3.093	0.002 **
Interpersonal sensitivity	1.39 ± 0.85	1.02 ± 0.71	4.238	<0.001 **
Depression	1.48 ± 0.85	1.09 ± 0.76	4.548	<0.001 **
Anxiety	1.24 ± 0.82	0.88 ± 0.71	4.371	<0.001 **
Hostility	1.12 ± 0.81	0.73 ± 0.59	4.844	<0.001 **
Phobic anxiety	0.91 ± 0.80	0.81 ± 0.76	1.319	0.187
Paranoid ideation	1.31 ± 0.84	0.85 ± 0.71	5.517	<0.001 **
Psychoticism	1.05 ± 0.77	0.66 ± 0.66	5.091	<0.001 **
Global Severity Index	1.27 ± 0.69	0.98 ± 0.64	4.104	<0.001 **

In Table 6, educated participants had significantly higher mental well-being scores in the depression dimension (Z=2.293; p=0.022) and paranoid ideation dimension (Z=2.431; p=0.015). Other SCL-90 dimensions and global severity index did not differ significantly when compared to the educational level group (p>0.05).

**Table 6:** Differences in mental well-being according to educational level (n=387) § P-value has been calculated using the Mann-Whitney Z-test. ** Significant at the p<0.05 level

SCL-90 variables	High school or below Mean ± SD	Bachelor or higher Mean ± SD	Z-test	P-value ^§^
Somatization	1.12 ± 0.79	1.08 ± 0.69	0.021	0.983
Obsessive-compulsive	1.25 ± 0.71	1.25 ± 0.70	0.100	0.921
Interpersonal sensitivity	1.08 ± 0.72	1.19 ± 0.81	0.985	0.325
Depression	1.07 ± 0.74	1.30 ± 0.83	2.293	0.022 **
Anxiety	0.96 ± 0.81	1.05 ± 0.77	1.293	0.196
Hostility	0.79 ± 0.72	0.92 ± 0.72	1.639	0.101
Phobic anxiety	0.87 ± 0.77	0.84 ± 0.78	0.434	0.664
Paranoid ideation	0.86 ± 0.72	1.09 ± 0.82	2.431	0.015 **
Psychoticism	0.73 ± 0.71	0.84 ± 0.74	1.011	0.312
Global Severity Index	1.02 ± 0.67	1.12 ± 0.67	1.341	0.180

In Table 7, the mental well-being mean scores of somatization, obsessive-compulsive, interpersonal sensitivity, depression, anxiety, hostility, phobic anxiety, paranoid ideation, psychoticism, and global severity index were not significantly different between the employed and unemployed (all p>0.05).

**Table 7:** Differences in mental well-being according to employment status (n=387) § P-value has been calculated using the Mann-Whitney Z-test.

SCL-90 variables	Employed Mean ± SD	Unemployed Mean ± SD	Z-test	P-value ^§^
Somatization	1.10 ± 0.69	1.07 ± 0.73	0.536	0.592
Obsessive-compulsive	1.24 ± 0.70	1.26 ± 0.70	0.368	0.713
Interpersonal sensitivity	1.21 ± 0.81	1.15 ± 0.78	0.848	0.396
Depression	1.26 ± 0.87	1.25 ± 0.79	0.009	0.992
Anxiety	1.07 ± 0.79	1.00 ± 0.77	0.862	0.389
Hostility	0.89 ± 0.67	0.89 ± 0.75	0.718	0.473
Phobic anxiety	0.81 ± 0.77	0.88 ± 0.78	1.054	0.292
Paranoid ideation	1.09 ± 0.81	1.01 ± 0.79	1.006	0.315
Psychoticism	0.83 ± 0.75	0.81 ± 0.73	0.073	0.942
Global Severity Index	1.12 ± 0.69	1.09 ± 0.67	0.458	0.647

In Table 8, the mean scores of SCL-90 symptom dimensions and global severity index did not differ significantly between living in the Central region or living in the Western region (all p>0.05).

**Table 8:** Differences in mental well-being according to residence location (n=387) § P-value has been calculated using the Mann-Whitney Z-test.

SCL-90 variables	Central region Mean ± SD	Western region Mean ± SD	Z-test	P-value ^§^
Somatization	1.06 ± 0.70	1.13 ± 0.73	0.675	0.500
Obsessive-compulsive	1.22 ± 0.71	1.33 ± 0.69	1.642	0.101
Interpersonal sensitivity	1.13 ± 0.79	1.25 ± 0.79	1.503	0.133
Depression	1.21 ± 0.81	1.34 ± 0.83	1.456	0.145
Anxiety	0.98 ± 0.77	1.12 ± 0.78	1.914	0.056
Hostility	0.86 ± 0.72	0.95 ± 0.71	1.403	0.161
Phobic anxiety	0.81 ± 0.77	0.94 ± 0.79	1.750	0.080
Paranoid ideation	0.99 ± 0.77	1.14 ± 0.85	1.488	0.137
Psychoticism	0.79 ± 0.73	0.87 ± 0.75	0.818	0.414
Global Severity Index	1.06 ± 0.67	1.18 ± 0.68	1.604	0.109

## Discussion

Mental health has a vital role in shaping our general well-being. Health is defined as a state of physical, social, and mental well-being, not merely the absence of disease [1]. Multiple factors could contribute to mental wellness. One of the possible examples is the rapid and unprecedented social change implemented during the COVID-19 pandemic. For instance, curfews and restrictions during the pandemic lead to limited social interactions. Another example is restrictions of physical activity associated with lockdown and the implementation of remote working. The increased stress of COVID-19, combined with the increased workload, administrative tasks, and increased charting in recent years, may contribute to the already high rates of burnout, suicide, and suicidal ideation [7]. The cumulative effect of various changes may lead to the detriment of the general population’s mental well-being. In addition, it is noted that there is an increase in obsessions related to health concerns brought forth by the COVID-19 pandemic, as any repetitive behavior increases obsessions. For instance, patients may display obsession related to excessive hand washing and fear of contamination. This may be explained by the added psychosocial stressors of the global pandemic [8].

Other researchers have researched mental well-being among the different populations. For instance, a study conducted over multiple countries to assess mental health during the COVID-19 lockdown with a sample of 418 participants found that males and females had significantly different levels of depression and anxiety [9]. In contrast to our study, the results were different, as there were no statistically significant differences in any domain of mental well-being (including depression and anxiety) among males and females. However, females had higher hostility and phobic anxiety levels than males (P = 0.065 and 0.077, respectively) although these findings are not statistically significant. Another local study, including the academic community in King Saud University (KSU), also found no significant differences between males and females in mental wellness [10]. Another local study, including Saudi females’ mental well-being, was conducted [11]. It showed a higher level of abnormal mental status in overweight participants and the oldest age group (> 25 years old). In contrast to our study, older patients (> 40 years old) had better mental well-being compared to younger patients (P < 0.001).

Furthermore, a study involving the general Saudi population found that female gender, chronic illnesses, smoking, and working in the private sector were significantly associated with mental illness [12]. In another study involving the general Saudi population, preventative measures like social distancing and handwashing were protective against anxiety, depression, and stress symptoms [13]. The same study found that high-schoolers, those who had poor self-reported health, and importantly healthcare workers had high Impact of Event Scale-Revised (IES-R) scale and Depression Anxiety Stress Scales (DASS). In our population, participants with a higher level of education had higher depression scores (P = 0.022).

Further use of the SCL-90 survey should be to assess general well-being in primary settings. This will ensure future researchers have a vast local body of literature and database as a reference point for comparison against the global body of literature.

One strength of our study was the survey utilized. The SCL-90 is a reliable and validated assessment tool. The study population was inclusive of participants from all socio-economic strata of different Saudi regions. The assessment of mental well-being is relevant to clinicians such as psychiatrists, clinical psychologists, and family physicians. Another benefit is to establish a reference for future researchers who wish to use the SCL-90 tool. Weaknesses in our study include the use of an online survey in place of physical questionnaires. Due to the nature of the COVID-19 curfew, we were not able to conduct in-person interviews. We included only patients 18 years or older, and the participants were not asked to fill the survey again after a two-week interval to reassess their mental well-being again. Due to the limited reach of the questionnaire and short time period, the population included in the study was not large.

## Conclusions

General mental well-being is a vital column of the biopsychosocial model and warrants further attention. Thus, we decided to assess the general mental well-being in the fallout of the COVID-19 pandemic. In our study, older participants had superior mental well-being in all domains except for two, phobic anxiety and somatization. Interestingly, married individuals had similar results in all domains except for two, and they were the same domains (phobic anxiety and somatization). The current status of the local vault of literature assessing the general mental well-being during the pandemic is deficient, pointing to a noticeable need for further local research.
